# Open and shut: Apoplastic water availability dominates stomatal immunity in determining disease resistance

**DOI:** 10.1093/plphys/kiaf176

**Published:** 2025-05-09

**Authors:** Josephine H R Maidment, Bo Xu

**Affiliations:** Assistant Features Editor, Plant Physiology, American Society of Plant Biologists; PHIM Plant Health Institute, Univ Montpellier, INRAE, CIRAD, Institut Agro, IRD, 34980 Montpellier, France; Centre de Biologie Structurale, INSERM, CNRS, Université de Montpellier, 34090 Montpellier, France; Assistant Features Editor, Plant Physiology, American Society of Plant Biologists; ARC Centre of Excellence in Plants for Space, and School of Agriculture, Food and Wine, Waite Research Institute, Glen Osmond, SA 5064, Australia

The physiological processes that balance water loss with water availability are core to plant function. Stomata are small pores on the surface of aerial plant tissues flanked by a pair of specialized guard cells. Water loss depends mainly on the opening and closure of stomatal pores, which controls the rate of water vapor diffusion from the leaf airspace into the atmosphere, but water loss is also affected by water availability in the leaf apoplast (Scharwies and Dinneny 2019; Melotto et al. 2024). Stomatal regulation, modulated by ion flux across surrounding guard cell pairs, optimizes plant water use, nutrient uptake, and carbon fixation and is responsive to environmental cues (Scharwies and Dinneny 2019).

Pathogenic microbes, such as *Pseudomonas syringae* pv. tomato (*Pst*), exploit these pores to invade plants, making stomata a battleground between host defense and pathogen attack (Hou et al. 2024; Melotto et al. 2024). Recognition of pathogen-associated molecules, such as the flagellin epitope flg22, activates immune responses to close stomata—a process termed stomatal immunity. However, *Pst* can overcome this defense by secreting coronatine, a jasmonic acid (JA) mimic. Coronatine acts on JA signaling pathways to reopen stomata for bacterial entry into the leaf apoplast (Melotto et al. 2024). Later in the infection process, *Pst* promotes apoplastic hydration by secreting effectors that manipulate abscisic acid (ABA) signalling to close stomata. This process, known as water soaking, creates an intercellular aqueous space that promotes bacterial colonization. In response, plants secrete defense peptides and activate salicylic acid signaling, attenuating *Pst-*induced ABA signaling and reopening stomata. This secondary immune response reduces water availability in the leaf apoplast and water soaking and is known as water immunity (Hou et al. 2024; Jian et al. 2024). However, the relative contributions of stomatal immunity and water immunity to disease resistance have remained unclear.

In a recent study published in *Plant Physiology*, Kemppinen et al. (2025) reported that water immunity overrides stomatal immunity for *Arabidopsis thaliana* resistance to *Pst* DC3000. To untangle the relative contributions of stomatal immunity and water immunity to disease resistance, the authors compared the disease outcome from spray inoculation—which requires bacterial entry through stomata and is affected by stomatal immunity—with syringe infiltration, which bypasses stomatal entry by directly introducing bacteria into the leaf apoplast. The authors studied stomatal apertures, extent of water soaking, and bacterial growth across a diverse panel of *A. thaliana* Col-0 mutants with defects in stomatal regulation at both early (1 h post inoculation) and later (24 h post inoculation) stages of infection.

The *ht1-2* (HIGH LEAF TEMPERATURE 1, HT1) mutant has constitutively closed stomata (Hõrak et al. 2016) and showed increased resistance to *Pst* following spray inoculation (Kemppinen et al. 2025). While this could reflect enhanced stomatal immunity, the authors showed that the *ht1-2* mutant also displayed reduced water soaking and lower *Pst* bacterial growth compared to the wild-type (WT) plants after syringe infiltration. This suggests that stomatal immunity alone does not explain the increased disease resistance observed in the *ht1-2* mutant. Furthermore, this indicates that factors other than stomatal dynamics affect the extent of water soaking and water immunity (Figure).

**Figure.**
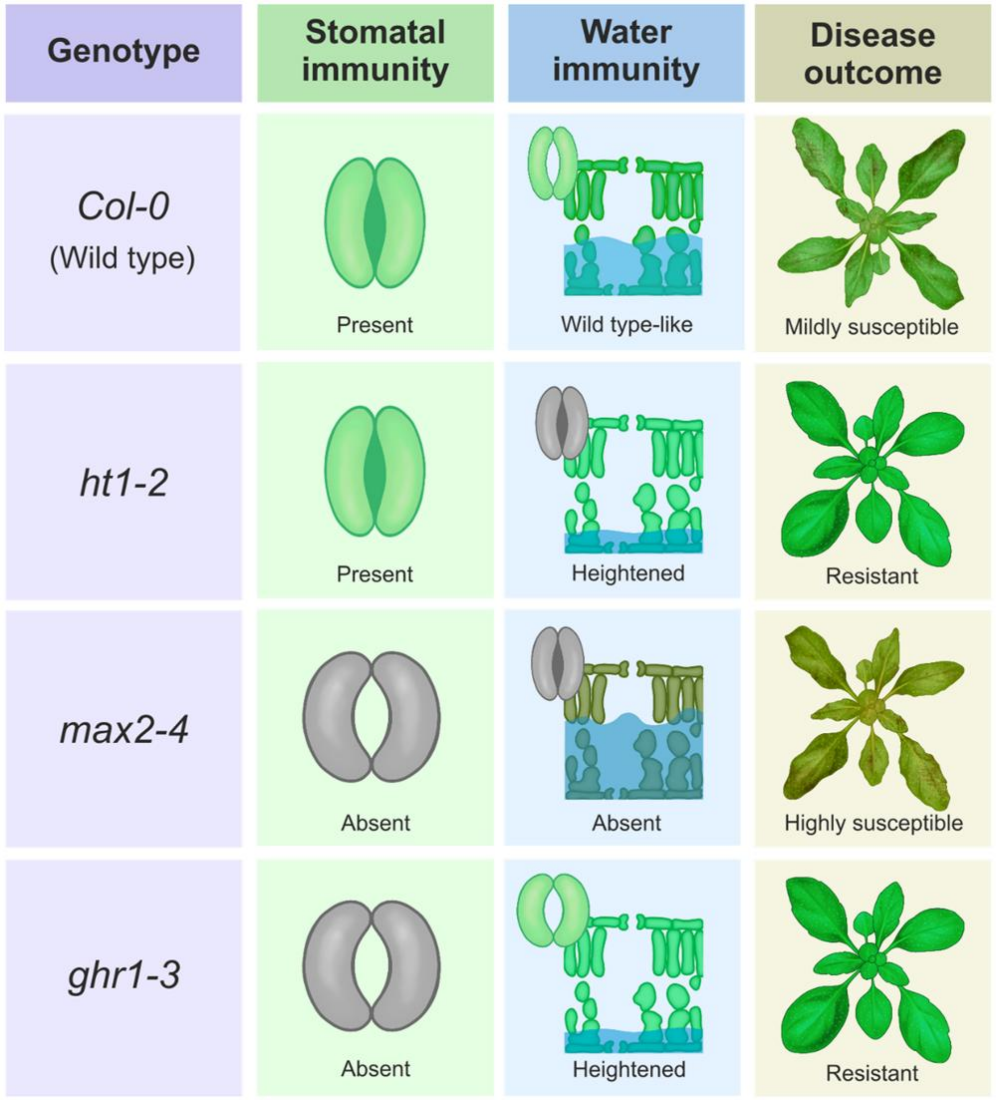
Adapted from Figure 4 in Kemppinen et al. (2025). Graphical summary of stomatal behavior, apoplastic hydration, and disease outcome for *A. thaliana* Col-0 (wild type) and the *ht1-2*, *max2-4* and *ghr1-3* mutants. Grey color indicates impaired stomatal regulation. The water level in blue shading in the water immunity column reflects the extent of apoplastic water availability (water soaking) during *Pst* infection.

The *max2-4* (MORE AXILLARY GROWTH2, MAX2) mutant has previously been shown to have impaired stomatal closure and increased susceptibility to *P. syringae* following spray-inoculation or syringe-infiltration (Piisilä et al. 2015). Consistent with this, Kemppinen et al. reported a lack of stomatal immunity in this mutant. However, this mutant exhibited increased water soaking, compared to WT plants, at the later stage of *Pst* infection by spray-inoculation, indicating compromised water immunity in this mutant. This was further supported by syringe infiltration experiments, where the *max2-4* mutant had more bacterial growth compared to WT plants. Overall, Kemppinen et al. show that both stomatal immunity and water immunity could contribute to the *Pst* disease outcome in this mutant (Figure).

Similarly to *max2-4*, the *ghr1-3* (GUARD CELL HYDROGEN PEROXIDE RESISTANT1, GHR1) mutant (Sierla et al. 2018) also has impaired stomatal immunity, with more open stomata compared to WT plants 1 h after spray-inoculation with *Pst* (Kemppinen et al. 2025). However, in contrast to *max2-4*, the *ghr1-3* mutant showed greater stomatal opening and less water soaking at 24 h post inoculation, indicating heightened water immunity compared to WT plants (Figure). Significantly, the *ghr1-3* mutant showed enhanced resistance to *Pst* relative to WT plants. This highlights that water immunity overrides stomatal immunity and dominates disease resistance (Kemppinen et al. 2025).

*Pst* manipulates host stomatal dynamics for opposing outcomes at different stages of infection by exploiting different hormone signaling pathways to promote either stomatal opening or closure (Melotto et al. 2024). Other phytopathogens also promote opposing plant physiological responses at different stages in host colonization; hemibiotrophic fungi, for example, have both a biotrophic and necrotrophic phase (Lee and Rose 2010). The current study highlights the importance of considering the temporal nature of pathogenesis when designing experiments. The authors also emphasize the importance of careful reporting of experimental conditions and methods. Physiological responses depend on integration of multiple environmental cues, and variation in factors such as light and humidity can accentuate or mask disease phenotypes.

Most of the mutants tested by Kemppinen et al. (2025) were defective in ABA-stimulated stomatal closure, except the *ht1-2* and *max2-4* mutants, whose stomatal regulation operates parallel to ABA signaling (Piisilä et al. 2015; Pantoja 2021). HT1 functions independently of ABA but shares targets with ABA downstream components, such as SLOW ANION CHANNEL 1 (SLAC1) (Hõrak et al. 2016). In contrast, MAX2 perceives strigolactones (Piisilä et al. 2015), raising the intriguing possibility that perception of strigolactones is linked to water immunity.

Overall, Kemppinen et al. (2025) shed light on the relative contributions of stomatal immunity and water immunity in controlling disease caused by *Pst*. They demonstrate that water immunity, rather than stomatal immunity, is the main determinant of disease progression. Whether the relative contributions of stomatal immunity and water immunity to the disease outcome are the same in other plant species, and for other pathogens which also rely on the establishment of an aqueous space for proliferation, remains to be determined. However, this study provides a useful methodological framework to explore other pathosystems. Understanding the role of stomata in disease and resistance is particularly important in the context of a changing climate and changing water availability.

## Data Availability

No new data were generated or analyzed in support of this research.
